# Prediction, Diversity, and Genomic Analysis of Temperate Phages Induced From Shiga Toxin-Producing *Escherichia coli* Strains

**DOI:** 10.3389/fmicb.2019.03093

**Published:** 2020-01-21

**Authors:** Yujie Zhang, Yen-Te Liao, Alexandra Salvador, Xiaohong Sun, Vivian C. H. Wu

**Affiliations:** ^1^College of Food Science and Technology, Shanghai Ocean University, Shanghai, China; ^2^Produce Safety and Microbiology Research Unit, U.S. Department of Agriculture, Agricultural Research Service, Western Regional Research Center, Albany, CA, United States

**Keywords:** Shiga toxin-producing *E. coli*, prophages, Stx-converting phage, induction, *stx* gene transfer

## Abstract

Shiga toxin-producing *Escherichia coli* (STEC) is a notorious foodborne pathogen containing *stx* genes located in the sequence region of Shiga toxin (Stx) prophages. Stx prophages, as one of the mobile elements, are involved in the transfer of virulence genes to other strains. However, little is known about the diversity of prophages among STEC strains. The objectives of this study were to predict various prophages from different STEC genomes and to evaluate the effect of different stress factors on Stx prophage induction. Forty bacterial whole-genome sequences of STEC strains obtained from National Center for Biotechnology Information (NCBI) were used for the prophage prediction using PHASTER webserver. Eight of the STEC strains from different serotypes were subsequently selected to quantify the induction of Stx prophages by various treatments, including antibiotics, temperature, irradiation, and antimicrobial agents. After induction, Stx1-converting phage Lys8385Vzw and Stx2-converting phage Lys12581Vzw were isolated and further confirmed for the presence of *stx* genes using conventional PCR. Phage morphology was observed by transmission electron microscopy. The prediction results showed an average of 8–22 prophages, with one or more encoding *stx*, were predicted from each STEC genome obtained in this study. Additionally, the phylogenetic analysis revealed high genetic diversity of Stx prophages among the 40 STEC genomes. However, the sequences of Stx prophages in the genomes of STEC O45, O111, and O121 strains, in general, shared higher genetic homology than those in other serotypes. Interestingly, most STEC strains with two or more *stx* genes carried at least one each of Stx1 and Stx2 prophages. The induction results indicated EDTA and UV were the most effective inducers of Stx1 and Stx2 prophages of the 8 selected STECs, respectively. Additionally, both Stx-converting phages could infect non-pathogenic *E. coli* (WG5, DH5α, and MG1655) and form new lysogens. The findings of this study confirm that Stx prophages can be induced by environmental stress, such as exposure to solar radiation, and lysogenize other commensal *E. coli* strains.

## Introduction

Shiga toxin-producing *Escherichia coli* (STEC) has been associated with numerous foodborne outbreaks around the world and causes severe illnesses, such as hemorrhagic colitis, bloody diarrhea, and hemolytic-uremic syndrome (Nüesch-Inderbinen et al., 2018). STEC can be easily disseminated and can cause human illness through direct contact with animal feces, contaminated irrigation water, and fecal-oral contamination of food items (Guy et al., 2014; Colello et al., 2016; Kennedy et al., 2017; Probert et al., 2017; Browne et al., 2018). *E. coli* O157:H7 was the first STEC strain discovered and was associated with contaminated burger patties in 1982; it has recently been related to leafy green outbreaks in multiple states (Juska et al., 2000; CDC, 2018). In addition, the number of infections caused by the top 6 non-O157 STEC strains – the serotypes O26, O45, O103, O111, O121, and O145 – has recently significantly increased (Marder et al., 2018). These non-O157 STEC strains can cause human illnesses as severe as the illnesses caused by *E. coli* O157:H7 and have been associated with numerous foodborne outbreaks around the world (Muniesa et al., 2004; Thomas et al., 2017; Nüesch-Inderbinen et al., 2018). The contamination of these pathogens, such as the serotypes O103, O26, and O121, has been associated with different types of food products, including meat, produce, and flour (CDC, 2019).

Many bacterial pathogens harbor virulence genes on plasmid or prophage sequences that are commonly associated with the transfer of virulence factors, also known as mobile genetic elements, among bacteria in the environment (Osińska et al., 2018). Shiga toxin (Stx) 1 and 2 are the major virulence factors of STEC strains contributing to the development of hemolytic-uremic syndrome (Bonanno et al., 2015; Dallman et al., 2015; Karmali, 2017). Numerous studies indicate that *stx* genes – *stx1* or *stx2* – encoding Stx are located in the sequence of a lambdoid prophage that can be induced from the bacterial genome by the SOS response and released into the environment as Stx-converting phages (Neely and Friedman, 1998; Schmidt, 2001). Stx-converting phages, also known as temperate phages, can infect susceptible bacteria through the lysogenic cycle and merge their genetic components into the bacterial chromosome (Chen et al., 2017). In the 2011 outbreak in Germany, a new pathogenic *E. coli* O104:H4 strain was identified and associated with contaminated sprouts, causing a total of 3816 infections and 54 deaths. The *E. coli* O104:H4 strain, originally characterized as enteroaggregative *E. coli* (EAEC), was found to harbor the *stx2a* gene and contain virulence properties from both EAEC and enterohemorrhagic *E. coli* (EHEC) (Frank et al., 2011). The emergence of this novel pathogen is closely related to the acquisition of the virulence factor *stx* via Stx-converting phages. Recently, several studies have shown that prophages were commonly found in the genomes of different bacterial species, such as *Clostridiales*, *Listeriaceae*, *Vibrio*, and *Enterobacteriaceae* (Chen et al., 2017; Zeng et al., 2017; Fortier, 2018). These prophages were highly associated with bacterial pathogenicity, such as toxins, secretion systems, and adhesions. Previous studies indicated that several virulence factors, such as RTX toxins, collagenases, lipases, agglutination, and aerolysin, were found in prophage-like sequences in most *Vibrio* species (Castillo et al., 2018). Nevertheless, similar studies with respect to STEC are lacking.

Free Stx-converting phages have been found in different environmental sources, such as wastewater, rivers, soil, and food (Grau-Leal et al., 2015). Additionally, 7.6 and 68.4% of all environmental samples tested were positive for Stx1-converting phages and Stx2-converting phages, respectively. Other studies have confirmed that Stx-converting phages could be induced from STEC strains under different stress conditions, including mitomycin C, ciprofloxacin, hydrogen peroxide, and UV irradiation, via the SOS response (Aertsen et al., 2005; Łoś et al., 2009, 2010; Imamovic and Muniesa, 2012; Allué-Guardia et al., 2013). Furthermore, some Stx-converting phages, such as bacteriophage 933W, have been demonstrated to be self-induced from strains without the presence of external stress (Plunkett et al., 1999). These findings likely indicate that Stx-converting phages could be induced from the STEC genomes, remain stable in the environment as potential genetic mobile elements, and distribute *stx* genes among bacterial populations.

Although the *stx* virulence genes have been found to be located on prophage sequences in STEC genomes, little is known about the association between different prophages and virulence genes in STEC strains. Prophages, as the critical mobile elements related to carrying virulence-related genes, have been investigated in bacterial species. However, information about the prevalence of different prophages other than Stx prophages in STEC is limited. Therefore, the objectives of this study were to predict various prophages from different STEC genomes and to evaluate induction and transduction capability of Stx prophages. The findings of this study serve as a foundation for further exploration of the ecology and evolution of STEC in the environment.

## Materials and Methods

### Prophage Prediction and Analysis

Forty complete genome sequences of different STEC strains, including the serotypes of O26, O45, O103, O111, O121, O145, and O157, were obtained from the National Center for Biotechnology Information (NCBI) GenBank database and are summarized in Supplementary Table S1. These 40 strains were initially isolated from creek sediment, water, and feces. PHASTER webserver was used to predict putative prophage sequences in the bacterial genomes (Arndt et al., 2017). Subsequently, all intact prophage sequences, including Stx prophages, were extracted from the bacterial genome sequences using Geneious software (version 11.1.5). The MAFFT algorithm was used to align 54 Stx prophage sequences in Geneious with default settings (Katoh and Standley, 2013). The phylogenetic analysis of the different Stx prophages was conducted using MEGA X with the neighbor-joining method and 1,000 bootstrap replicates. The phylogenetic tree was subsequently displayed using the Interactive Tree Of Life (iTOL) online tool (Kumar et al., 2016; Letunic and Bork, 2019).

### Stx Prophage Induction

Among the 40 STEC strains, eight, RM8246, RM11911, RM8385, RM9975, RM8385, RM12581, EDL933, and RM9245, were selected and obtained from the U.S. Department of Agriculture (USDA), Agricultural Research Service (ARS), and Western Regional Research Center (WRRC) for the experiment of Stx prophage induction. These eight strains were initially isolated from the environment. Fresh cultures were prepared by inoculating 10 ml of trypticase soy broth (TSB, Difco, Becton Dickinson, Sparks, MD, United States) with a loopful of frozen cultures and incubated at 37°C overnight with shaking at 175 rpm before use.

Various treatments, including antibiotics, temperature, irradiation, and antimicrobial agents, were used as stress factors to determine the induction efficiency of Stx prophages from the eight selected STEC strains. An aliquot of 10 ml bacterial culture of each STEC strain was incubated at 37°C until the OD_600_ reached 0.35–0.4 and was subsequently treated with different concentrations of mitomycin C (0.5 and 1 μg/ml) and ciprofloxacin (0.4 and 4 μg/ml), EDTA (20 mM, pH 8.5), ClO_2_ (0.5 ppm), different temperatures (28 and 37°C as a control), and UV intensity (55 and 110 J/m^2^). The treated cultures were incubated at 37°C for 18 h for Stx prophage induction. After incubation, the induced phages were obtained by centrifugation at 5,000 × *g* for 10 min and filtration through 0.22 μm sterile membrane filters. The experiment for prophage induction of each strain under different induction conditions was repeated in three replications. The phage lysates were later used for both the quantification and isolation of Stx-converting phages.

### Quantification of Stx-Converting Phages Using qPCR

The induced phages were quantified based on the *stx* genes using qPCR. The phage lysates were treated with DNase I (100 U/ml of phage lysate) at 37°C for 1 h prior to phage DNA extraction. Phage DNA was obtained using a phage DNA extraction kit (Norgen Biotek Corp., Thorold, ON, Canada) according to the manufacturer’s instructions. Before the experiment, the *stx* gene sequences of selected strains were obtained and subjected to BLASTn search for the specificity of primers. All primers and probes used in this study are shown in Supplementary Table S2 (Integrated DNA Technologies, Coralville, IA, United States). The qPCR procedures were performed as previously described by Grau-Leal et al. (2015) with modifications. Briefly, the *stx1* and *stx2* standards were constructed by inserting a 277-bp fragment of *stx1* and a 378-bp fragment of *stx2*, both amplified from *E. coli* strain RM7190, into the plasmid pGEM-T, respectively. Subsequently, the inserted *stx* sequences were confirmed by Sanger sequencing prior to quantification. A 20-μl reaction volume was used for the amplification of target genes using real-time PCR (Bio-Rad CFX96, United States). The reaction mixture contained 10 μl of PCR master mix (2×), 0.4 μl of *stx1*/*stx2* probe (10 nM), 0.6 μl each of the *stx1*/*stx2* forward and reverse primers (10 nM), 3.4 μl of distilled H_2_O, and 5 μl of DNA. The thermal cycling parameters for denaturing, annealing, and extension of the *stx* genes were 95°C (3 min), 95°C (15 s), and 60°C (1 min), respectively, for 40 cycles. All samples, including standards and negative controls, were run in triplicate. The gene copy (GC) number was calculated for the quantification of Stx prophage induction based on the standard curve and presented as an average of three replications.

### Isolation of Purification of Induced Phages

The phages obtained from the highest induction level according to qPCR were subjected to isolation and purification. Each phage lysate (*n* = 8) was spotted on no-salt, soft top agarose (NS-ST, 10 g bacto tryptone, 5 g yeast extract, 4 g agarose, 10 mM MgSO_4_/liter) pre-mixed with *E. coli* WG5 strain and incubated at 37°C for 18 h to determine phage infectivity (Larrañaga et al., 2018). Subsequently, the phages were further purified using a single-plaque purification method on WG5 as previously described with slight modifications (Liao et al., 2019). In brief, plates containing 10 ml of bottom Luria-Bertani agar (LB, Difco, Becton Dickinson) were prepared in advance. An aliquot of 50 μl of the phage was mixed with 100 μl of *E. coli* WG5 in 5 ml of NS-ST agarose and poured on LB agar plates, followed by incubation at 37°C for 18 h. Random single plaques were picked from each sample for further purification. After single phage purification for three times, the purified phages were propagated using *E. coli* WG5, and the bacterial debris was removed by centrifugation at 5,000 × *g* for 10 min and filtration through sterile 0.22-μm membrane filters. Phage lysates were concentrated using 50 KDa Amicon Ultra filter columns (Merck Millipore, Schwalbach, Germany) and subsequently subjected to CsCl gradient centrifugation at 131,300 × *g* for 24 h. The CsCl-treated phages were resuspended in SM buffer and then treated with DNase I (100 U/ml of phage lysates) at 37°C for 1 h to remove bacterial DNA from the phage lysates. The treated phages were then used for morphological classification via electron microscopy and for the screening of the *stx* genes using conventional PCR.

### PCR Screening of the *stx* Genes of the Stx-Converting Phages

DNA was extracted from the purified phages using a phage DNA extraction kit (Norgen Biotek Corp., Thorold, ON, Canada) according to the manufacturer’s instructions. Screening of the *stx* genes encoded in the Stx-converting phages was conducted using conventional PCR with a reaction volume of 25 μl. The primers used for amplifying the *stx1* and *stx2* genes are illustrated in Supplementary Table S2. The thermal cycling parameters of denaturing, annealing, and extension for the *stx1* and *stx2* genes were 95, 56, and 72°C and 95, 58.1, and 72°C, respectively, for 28 cycles.

### Transmission Electron Microscopy

A 6-μl aliquot of CsCl-treated phage lysate was placed on copper mesh PLECO grids (Ted Pella Inc., Redding, CA, United States) and incubated at room temperature (approximately 26°C) for 1 min. The phage-containing grids were blotted on Whatman filter paper and then subjected to negative staining by adding 4 μl of 0.75% uranyl acetate (Sigma-Aldrich, Darmstadt, Germany) for 10 s at room temperature (approximately 26°C). The grids were then examined for phage morphology under a transmission electron microscope (Tecnai G2 F20 model FEI, United States).

### Phage Transduction

Four laboratory *E. coli* strains, WG5, DH5α, C600, and MG1655, were used for phage transduction. A fresh overnight culture of each strain was prepared in LB broth and incubated at 37°C for 18 h. An aliquot of 100 μl of each culture was mixed with 5 ml of molten NS-ST agar and poured into a prepared plate containing 10 ml of bottom LB agar. A total of 10 μl of the isolated Stx-converting phages were spotted on the plate mixed with each strain, which was subsequently incubated at 37°C for 18 h. After incubation, the colonies in the clear lysis zone of each plate were picked and streaked on LB plates. The plates were incubated at 37°C for 18 h. To avoid the contamination of the Stx-converting phages, a single colony was picked and streaked on LB plates. The plates were incubated again at 37°C for 18 h. The procedures were repeated three times. The presence of the *stx* genes in the final colony was confirmed using conventional PCR as described in the previous section.

## Results

### Prediction of Prophage Sequences in STEC Strains

The results of the PHASTER prediction showed that approximately 8–22 different prophage sequences were predicted in each genome of the selected STEC strains (Table 1). These prophages contained genome sizes ranging from 5.6 to 131.9 kb or GC contents ranging from 40.8 to 57.8%. Additionally, 75.4% of the total predicted prophages among 40 STEC strains were intact, whereas 15.3 and 9.3% were incomplete and questionable, respectively. However, five *E. coli* O121 strains contained the highest percentage of intact prophage sequences, ranging from 86.7 to 92.85%, compared to the strains from other serotypes in this study. Furthermore, the most commonly identified prophages among the selected strains were closely related to the Stx1-converting bacteriophage *Enterobacteria* phage BP-4795 (Creuzburg et al., 2005) and were located in the region between 5.7 and 123.4 kb of the bacterial genomes, regardless of the strain. The second most commonly identified prophages were similar to *Enterobacteria* phage lambda, which contain a dsDNA genome 48,502 bp in size and exhibit the morphology of the *Siphoviridae* family (Chen and Richardson, 1987). This prophage sequence was located in the bacterial genome between the region of 10.8 and 112.8 kb among different positive strains. The next commonly identified prophages, located in the bacterial genome between 5.7 and 62.5 kb, were similar to the Stx2-converting phage 1717 obtained from an *E. coli* O157:H7 strain. The phage 1717 has a 62,147-bp dsDNA genome and belongs to the *Siphoviridae* family. The results of the study showed that most prophages predicted from the selected STEC strains belonged to the *Siphoviridae* family.

**TABLE 1:** Different prophage contents, including intact, questionable, and incomplete prophages, predicted from 40 STEC genomes obtained from NCBI using the PHASTER webserver^∗^.

Serotypes	Strains	Number of intact	Number of questionable	Number of incomplete
		prophages (size)	prophages (size)	prophages (size)
O26	RM10386	15 (14-115.2 kb)	2 (9.8-17.9 kb)	3 (12.4-33.3 kb)
	RM8426	16 (14-119.4 kb)	1 (9.8 kb)	1 (12.4 kb)
	2013C-3277	12 (27.9-96.6 kb)	n.d.	4 (7.9-46.4 kb)
	S17-13	6 (18.7-62 kb)	n.d.	2 (18.9-35.3 kb)
	11368	16 (14-114.9 kb)	2 (9.8-17.9 kb)	2 (12.4-16.3 kb)
O45	RM11911	12 (10.7-65.6 kb)	1 (50.7 kb)	1 (19.2 kb)
	2011C-4251	12 (20.8-109.1 kb)	n.d.	5 (10.8-44.6 kb)
	RM13745	10 (10.7-95.9 kb)	1 (50.7 kb)	1 (19.2 kb)
	RM13752	10 (10.7-96.1 kb)	1 (50.7 kb)	1 (19.2 kb)
	SJ7	9 (39.3-100.9 kb)	6 (14.7-58 kb)	2 (6.6-21.6 kb)
O103	RM8385	16 (14-79.1 kb)	3 (9.8-15.9 kb)	n.d.
	12009	11 (32.6-117.8 kb)	2 (10.2-13.5 kb)	4 (10.8-44.5 kb)
	2015C-3163	12 (29.3-116.8 kb)	2 (9.8-23.6 kb)	1 (17 kb)
	2013C-3264	16 (17-123.4 kb)	1 (29.9 kb)	4 (7.3-21.8 kb)
	2013C-4225	15 (14-79.1 kb)	2 (9.8-17.9 kb)	1 (9.8 kb)
O111	RM9975	9 (11.1-77 kb)	4 (8.5-40.2 kb)	1 (20.7 kb)
	11128	13 (11.1-68.4 kb)	n.d.	6 (5.8-38.8 kb)
	95JB1	13 (11.1-62.7 kb)	2 (17.6-38.2 kb)	6 (5.6-26.3 kb)
	95NR1	14 (11.1-131.9 kb)	2 (17.6-38.2 kb)	6 (5.6-26.3 kb)
	2015C-3101	11 (11.1-78.3 kb)	1 (40.1 kb)	3 (8.5-40.2 kb)
O121	RM8352	13 (20.1-113.7 kb)	1 (21.4 kb)	n.d.
	16-9255	13 (20.1-111.6 kb)	1 (23.6 kb)	1 (28.4 kb)
	2015C-3107	13 (21.5-111.4 kb)	2 (22.1-23.6 kb)	n.d.
	2014C-3599	13 (20.2-114.6 kb)	n.d.	1 (10.8 kb)
	2014C-4423	13 (15.2-112.8 kb)	n.d.	2 (23.3-25.9 kb)
O145	RM12581	19 (11-64.7 kb)	n.d.	2 (7.2-25.8 kb)
	95-3192	17 (11-66.4 kb)	2 (12.6-13 kb)	3 (7.2-37.2 kb)
	2014C-3084	13 (11-84.1 kb)	1 (13 kb)	3 (5.7-16.6 kb)
	2015C-3125	18 (11-110.9 kb)	1 (14.3 kb)	2 (8-16.6 kb)
	RM9872	17 (11-61.8 kb)	1 (13 kb)	2 (7.2-16.6 kb)
O157	EDL933	11 (24.8-113.5 kb)	3 (15.2-26.5 kb)	2 (9.6-17.5 kb)
	pv15-279	13 (23.3-109 kb)	4 (17.9-41.2 kb)	4 (10.8-22.7 kb)
	EC4115	13 (24.7-119 kb)	2 (20.6-31.6 kb)	2 (21.7-38 kb)
	TW14359	13 (26.1-98 kb)	2 (20.5-31.6 kb)	2 (29-38 kb)
	SS17	13 (24.7-103.6 kb)	1 (20.4 kb)	3 (10.9-21.7 kb)
Other serotypes	RM9245	10 (11.2-76.2 kb)	3 (10.8-46.6 kb)	2 (27.5-37.2 kb)
	2011C-3493	7 (17.4-63.6 kb)	1 (66.8 kb)	3 (21.7-28.7 kb)
	FDAARGOS_403	6 (17.5-68.7 kb)	1 (73.7 kb)	4 (21.7-28.7 kb)
	SMN197SH3	13 (16.1-109.8 kb)	2 (11.9-15.3 kb)	2 (5.7-18.4 kb)
	HUSEC2011	6 (22.3-68.7 kb)	2 (24.3-73.7 kb)	4 (7.9-28.7 kb)

*^∗^PHASTER analyses were conducted in August 2018. n.d: not detected.*

### Phylogenetic Analysis of Stx Prophages in STEC Strains

**FIGURE 1:**
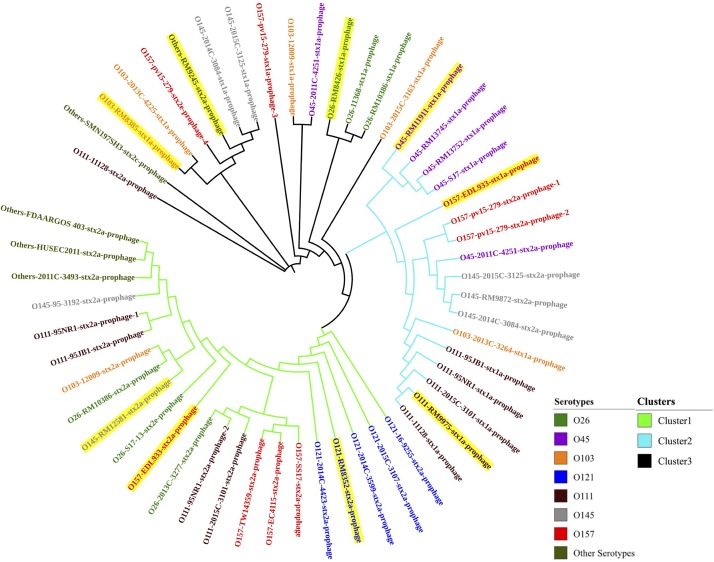
The neighbor-joining phylogenetic tree of Stx prophages predicted from 40 STEC genomes in this study. Bootstrap analysis was conducted using 1,000 replicates for reliability. Highlighted Stx prophages represent those predicted in the eight STEC strains selected for prophage induction.

Through PHASTER analysis, the results revealed that each *stx* gene predicted in the STEC genome fell within the region of a corresponding intact Stx prophage. A total of 54 Stx prophage sequences were extracted from the 40 STEC genomes and analyzed to determine their genetic variety. The phylogenetic analysis showed that three distinct phage clusters, designated Cluster 1, Cluster 2, and Cluster 3, were identified (Figure 1). Each cluster contained Stx prophages obtained from more than five different STEC serotypes. Twenty-two Stx2 prophages classified in Cluster 1 were detected among most STEC serotypes except O45. Cluster 2 was composed of 11 Stx1 prophages and six Stx2 prophages, of which the Stx2 prophages shared higher sequence similarity with other Stx2 prophages than with the Stx1 prophages. Additionally, the prophages classified in Cluster 3 contained 12 Stx1 and three Stx2 prophages; these prophages were detected in most STEC serotypes except O121. The phylogenetic analysis demonstrated the high diversity of Stx prophages, for both Stx1 and Stx2 prophages, among different STEC strains.

Additionally, the prophages predicted within each serotype of the STEC strains were compared, and the phylogenetic results showed that the Stx prophages detected in the STEC O45, O111, and O121 strains were generally more conserved than those detected from other serotypes, such as O145 and O157 (Figure 1). This finding likely shows that some serotype of STEC strains, such as O157, are capable of accepting wide range of different Stx prophages, but others, like O121, are only susceptible for the infection of certain phages with very narrow genetic diversity.

In this study, most STEC strains with more than one *stx* gene, such as the STEC O26 strain (RM10386), STEC O103:H2 strain (12009), STEC O111 strain (11128), and STEC O157:H7 strain (EDL933), contained at least one of each of the Stx1 and Stx2 prophages. Interestingly, the phylogenetic results showed that 4 Stx prophages encoded in the genome of the *E. coli* O157:H7 strain (pv15-279) were classified into two different clusters (Clusters 2 and 3). In addition, 3 Stx prophages detected in the *E. coli* O111 strain (95NR1) were classified into 2 distinct clusters (Clusters 1 and 2). These results indicated the diversity of the Stx1 and Stx 2 prophages predicted from the same STEC strain.

### Induction of Stx Prophages by Different Stress Factors

**FIGURE 2:**
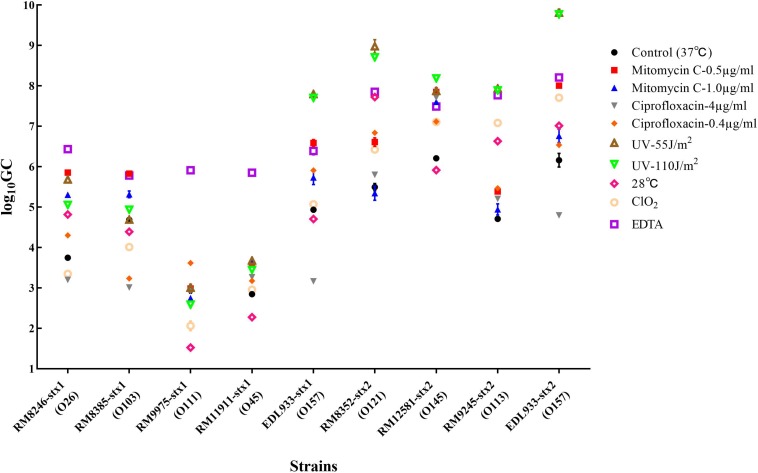
Stx-converting phages evaluated by qPCR in lysates generated from STEC strains without inducing agent (control) or with different treatments, 18 h after induction. The results are the average of three independent experiments. Error bars show SD.

Among the 40 STEC strains used for prophage prediction, 8 STEC strains were selected to evaluate the effects of various factors on the induction of Stx prophages. The results showed that without any treatment (control group), the selected STEC strains containing Stx1 and Stx2 prophages resulted in approximately 10^3^–10^5^ and 10^5^–10^7^ GC ml^–1^ of the induced phages, respectively (Figure 2). The results indicated that most STEC strains were able to self-induce and release Stx-converting phages into the environment. On the other hand, the results showed that most treatments used in this study were able to trigger prophage induction and to release Stx-converting phages. Among all treatment types, the maximum induction level of Stx-converting phages, with 10^10^ GC ml^–1^, was detected upon the treatment with UV intensity of 55 J/m^2^, demonstrating the high possibility of prophage induction in the environment. Regardless of phage type, EDTA and UV treatment were the most efficient inducing factors for Stx1-converting and Stx2-converting phages, respectively. Moreover, the results also showed that the levels of the induced Stx2-converting phages were generally higher than those of the induced Stx1-converting phages under the same treatment (Figure 2). Interestingly, the results showed that the *E. coli* O157:H7 strain (EDL933), containing Stx1 and Stx2 prophages, had the highest level of both types of phages induced compared to the other tested strains either by self-induction or by different treatments.

### Characterization of the Stx-Converting Phages

**FIGURE 3:**
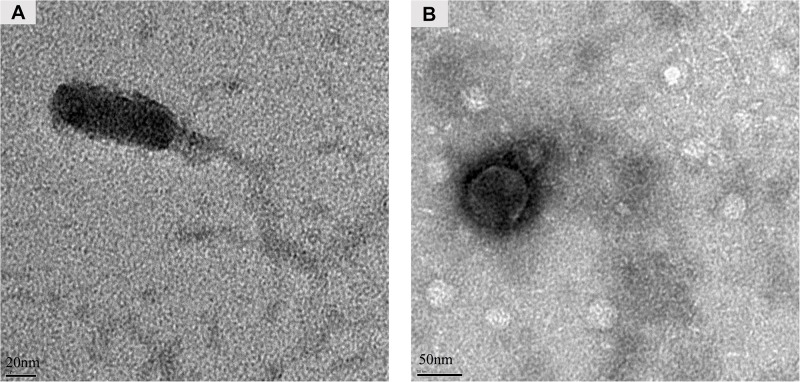
Morphology of Stx-converting phages observed by transmission electron microscopy. **(A)** Phage Lys8385Vzw with a long and non-contractile tail. **(B)** Phage Lys12581Vzw with a short tail composed of 6 short subterminal fibers.

The presence or absence of the *stx* genes in the randomly picked purified phages was further screened using conventional PCR. As a result, phage Lys8385Vzw was positive for the *stx1* gene and induced from *E. coli* O103:H11 strains (RM8385). Lys12581Vzw, containing one *stx2* gene, was induced from the O145:H28 strain (RM12581). For morphological classification, the Stx1-converting phage Lys8385Vzw had a capsid approximately 83.6 ± 0.5 nm in diameter and a long non-contractile tail 188.4 ± 0.5 nm in length, belonging to the *Siphoviridae* family (Figure 3A). Interestingly, some phages were found in which the tail was connected to the other capsid with 30 ± 0.5 nm in diameter (Supplementary Figure S1). On the other hand, the morphology of the Stx2-converting phage Lys12581Vzw was similar to that of the *Podoviridae* family. Lys12581Vzw had a capsid approximately 77 ± 0.5 nm in diameter and a short tail composed of 6 short subterminal fibers (Figure 3B).

### Transduction Test of the Stx-Converting Phages

After spotting the Stx-converting phages on NS-ST agar with a bacterial lawn of the WG5, DH5α, MG1655, and *E. coli* C600 laboratory strains, the surviving bacterial colonies in the lysis zone of the spot test plates were picked and further confirmed for the presence of the *stx* genes using conventional PCR. The results showed that the colonies from laboratory strains of WG5, DH5α, and MG1655 were susceptible to the infection with both the Stx1-converting phage Lys8385Vzw and Stx2-converting phage Lys12581Vzw and became positive for either the *stx1* or *stx2* gene, indicating that both Stx-converting phages were able to lysogenize the laboratory strains WG5, DH5α, and MG1655 (Table 2). However, similar results were not found with the C600 strain because no colony was positive for either the *stx1* or *stx2* gene by PCR.

**TABLE 2:** Confirmation of *stx* genes present the in the potential lysogens of *E. coli* WG5, MG1655, C600, and DH5α after infection with the selected Stx-converting phages through spot assay.

Stx-converting^∗^	Presence of *stx* gene^α^			
Phage	*E. coli* WG5	*E. coli* MG1655	*E. coli* C600	*E. coli* DH5α
Phage Lys8385Vzw	+	+	−	+
Phage Lys12581Vzw	+	+	−	+

***^∗^**Phage Lys8385Vzw and Lys12581Vzw contain contains *stx1* and *stx2* genes, respectively. ^α^Presence of *stx* genes in the recipient strains were determined using conventional PCR; + and − indicate positive and negative of *stx* genes, respectively.*

## Discussion

Shiga toxin-producing *Escherichia coli*, as one of the most common pathotypes of pathogenic *E. coli* strains, characteristically produces Stx and causes several human illnesses (CDC, 2014). A previous study demonstrated that the induction of Stx prophages was closely associated with the production of Stx and the transfer of *stx* genes among susceptible strains (Krüger et al., 2018). Although there is increasing interest in the prediction and functions of prophages found in different bacterial species, such as *Ochrobactrum* and *Brucella* (Hammerl et al., 2016; Jäckel et al., 2017), related information with respect to prophages in STEC genomes is lacking. In this study, the 40 complete whole-genome sequences of STEC strains generated by next-generation sequencing (NGS) technology and obtained from the NCBI database were used to understand the presence of different prophages within STEC genomes.

In the selection of STEC genomes from NCBI for prophage prediction, two different sequencing files, generated from whole-genome shotgun sequencing technology and NGS technology, were found for some strains, such as *E. coli* EDL933. Our preliminary PHASTER results showed that the *E. coli* EDL933 genome sequence obtained from NGS technology contained 11 intact prophages, 3 questionable prophages, and 2 incomplete prophages. However, the genome sequence from the shotgun sequencing technology resulted in only 1 intact prophage, 1 questionable prophage, and 6 incomplete prophage sequences (data not shown). Therefore, the bacterial genome sequences obtained from different sequencing platforms could lead to varying results in prophage predictions. The discrepancy likely results from the limitation of whole-genome shotgun sequencing, an older platform that acquires contiguous sequence data of large genomes and that involves genome assembly by finding overlapping fragments. It has several limitations, such as a time-consuming process, errors in genome alignment, and the challenge of assembling repetitive sequences in genomes (Karaman, 2003). Currently, NGS technology has been used for a broad range of applications due to the improvement of accuracy and sensitivity and has significantly facilitated the scope of genetic research (Metzker, 2010). These findings indicate that NGS technology allows us to accurately predict prophage sequences and to better understand the distribution of prophages in STEC genomes.

In this study, among all predicted prophages from STEC strains, the top three most common prophages, not necessarily Stx prophage but similar to Stx1-converting phage, were *Enterobacteria* phage BP-4795, *Enterobacteria* phage lambda, and Stx2-converting phage 1717 (Chen and Richardson, 1987; Creuzburg et al., 2005). The phage sequences contained a putative *int* gene, which was encoded in both *Enterobacteria* phage BP-4795 and Stx2-converting phage 1717 and has been demonstrated to be a critical factor associated with the integration of phage DNA into bacterial chromosomes through lysogenic infection among susceptible bacterial strains (Menouni et al., 2015). This fact may account for the high prevalence of the predicted prophages among different strains in this study.

Interestingly, in this study, most predicted Stx1 and Stx2 prophages from the 40 selected STEC genomes were closely associated with the Stx1-converting phage *Enterobacteria* phage BP-4795 and *Enterobacteria* phage 933W, respectively. Previous results have demonstrated that all Stx prophages were intact sequences in the bacterial genomes and posed potential for induction after external stress. A number of studies have indicated that most Stx2-converting phages induced from different STEC strains shared high genetic similarity, particularly the *stx* genes and *stx*-related regulatory genes, with *Enterobacteria* phage 933W (Yin et al., 2015; Krüger et al., 2018). Additionally, *Enterobacteria* phage 933W has a high frequency of self-induction from its host, which easily enables its distribution among bacterial populations (Allué-Guardia et al., 2013). Together with the current results, these findings account for the wide distribution of Stx prophage sequences among STEC strains, such as those predicted in this study, which are similar to the genomes of *Enterobacteria* phage BP-4795, *Enterobacteria* phage lambda, and Stx2-converting phage 1717. Additionally, these Stx prophages are considered mobile genetic elements and likely result in the potential transfer of virulence genes among different bacterial hosts.

In our study, the prediction results showed that most strains from the serotypes O111, O145, and O157 carried more than one Stx prophage. The integration of Stx prophages in the bacterial genomes is highly associated with a specific location called an insertion site. A previous study demonstrated that there were five common insertion sites associated with Stx prophages integrating into the host chromosome (Balding et al., 2005; Creuzburg et al., 2005; Karlsson et al., 2006; Serra-Moreno et al., 2007). Furthermore, the distribution of the occupied loci varies among different serotypes, which could also lead to the differences in the Stx prophages present in bacterial genomes. The current results showed that Stx prophages predicted in the STEC O145 and O157 strains in cluster 2 and 3 shared high similarity. Cooper et al. provided evidence regarding the same lineage of genetic evolution between *E. coli* O145 and O157 strains, indicating that both serotypes of strains contained similar core virulence factors, including Stx prophages (Cooper et al., 2014). In contrast, the current results showed that the sequences of Stx prophages were conserved within each serotype of O45 and O121 but were distinctive from the strains of different serotypes. Furthermore, only one Stx prophage, either Stx1 or Stx2, was detected among the STEC O45 and O121 strains in this study, and thus, the phenomenon was likely associated with limited insertion sites in the bacterial genome. These findings demonstrate that similar Stx prophages may prefer the same insertion sites and can be easily exchanged under stress.

The induction of Stx prophages from the STEC strains plays a key role in the distribution of *stx* genes through Stx-converting phages in the environment. In this study, several environmental stress factors, such as UV, temperature, antibiotics, and two antimicrobial agents commonly used in food-processing environments – EDTA and ClO_2_ – were evaluated for the capability of inducing Stx prophages. The current results showed that Stx2-converting phages were easily induced under most conditions, with UV treatment being the most effective inducing factor. Previous studies found that free Stx2-converting phages were more prevalent than Stx1-converting phages in the environment (Grau-Leal et al., 2015; Quirós and Muniesa, 2017). Their results were in agreement with our findings that Stx2-converting phages are more capable of induction by external stress than Stx1-converting phages. Furthermore, the results of this study demonstrated that EDTA was the most effective inducing factor for Stx1 prophage but was also capable of inducing Stx2 prophage. Although EDTA is a common antimicrobial agent used for food preservation (Gadang et al., 2008; Sivarooban et al., 2008), EDTA treatment may pose a risk of triggering the induction of either Stx1 or Stx2 prophage from STEC and of releasing the Stx-converting phages in the surrounding food-processing environment. For other parameters commonly used in the food industry, Aertsen et al. (2005) found that high pressure used in food processing had little effect on the induction of Stx2-encoding prophage from several environmental STEC isolates. Another study also indicated that heat treatment in food preservation was not able to induce Stx2 prophage from the *E. coli* O104:H4 strain, which was related to the 2011 outbreak in Germany (Fang et al., 2017). The authors also found that H_2_O_2_, typically used as an antimicrobial agent, was able to induce Stx prophage from the strain through the SOS response mechanism. Additionally, they found that both HCl and lactic acid could trigger the induction of Stx prophage via a different pathway other than the SOS response. Furthermore, Imamovic et al. (2009) confirmed that Stx-converting phages induced from the STEC strains were able to infect non-pathogenic *E. coli* and form a new lysogen through the transduction of *stx* genes; the results were in agreement with the findings of the current study that both Stx1- and Stx2-converting phages were able to infect and lysogenize most laboratory strains to form new lysogens with *stx* genes. These findings likely reveal that interventions for controlling STEC in food processing should be carefully selected to prevent Stx prophage induction.

The numbers of characterized Stx-converting phages from different STEC strains are relatively scarce compared to those of characterized lytic phages. Only 5 well-studied Stx2-converting phages, bacteriophage 933W, Stx2-converting phage II, *Enterobacteria* phage Min27, Stx2-converting phage vB_EcoP_24B, and *Escherichia* phage phi191, could be obtained from the International Committee on Taxonomy of Viruses (ICTV) database to date. All Stx2-converting phages were classified in the family *Podoviridae*. In this study, the morphology of Stx2-converting phage Lys12581Vzw also belongs to the family *Podoviridae* and was consistent with the morphology of Stx2-converting phage 933W (Yan et al., 2011). In addition, the whole-genome sequence of phage Lys12581Vzw revealed that the phage tail sequence of phage Lys12581Vzw shares high similarity with the phage tail sequences of other Stx2-converting phages (data not shown). This finding provides valuable insights into the diversity of Stx2-converting phages in the *Podoviridae* family. Furthermore, the Stx1-converting phage (Lys8385Vzw) induced in this study has two different morphologies; one was similar to the *Siphoviridae* family, and another has two capsids with a long tail which is different from other Stx1-converting phages belonging to *Siphoviridae* family, such as *Enterobacteria* phage BP-4795 (GenBank NC_004813) and phage phi-O153 (GenBank AY838795), and *Podoviridae* family, such as phage AU6Stx1 (GenBank KU977420) and AU5Stx1 (GenBank KU977419) (Creuzburg et al., 2005). To our knowledge, none of the published phages has similar morphology as to the unusual morphology of phage Lys8385Vzw. Further characterization of the phage Lys8385Vzw should be conducted in future studies.

To our knowledge, this is the first report on the distribution of various prophages, including Stx prophages, in different serotypes of STEC genomes. In this study, genomic analyses showed that Stx prophages were highly diverse among different strains, and the diversity of the prophages was related to the different STEC serotypes. Additionally, the genomically predicted Stx prophages were further confirmed with *in vitro* experiments, showing the inducibility of the prophages from the STEC hosts under certain environmental stress factors, such as UV irradiation. Most importantly, the Stx-converting phages released from the STEC strains were able to infect other *E. coli* strains and form new pathogens. These findings substantiate the potential risk of virulence gene transfer through the transduction associated with Stx prophages. Future studies will be explored to understand the ecology and evolution of STEC associated with prophages in the environment.

## Data Availability Statement

The raw data supporting the conclusions of this article will be made available by the authors, without undue reservation, to any qualified researcher.

## Author Contributions

YZ was responsible for phage prediction, phage induction and isolation, data analysis, and manuscript preparation. Y-TL was responsible for the manuscript preparation. AS was responsible for TEM. XS was responsible for assisting experiment design. VW conceived the study, aided in experiment design, and the manuscript preparation. All authors reviewed the manuscript.

## Conflict of Interest

The authors declare that the research was conducted in the absence of any commercial or financial relationships that could be construed as a potential conflict of interest.

## References

[B1] Aertsen A. Faster D. Michiels C. W. (2005). Induction of Shiga toxin-converting prophage in *Escherichia coli* by high hydrostatic pressure. *Appl. Environ. Microbiol.* 27 434–441. 10.1128/AEM.71.3.1155-1162.2005 15746313 PMC1065167

[B2] Allué-Guardia A. Imamovic L. Muniesa M. (2013). Evolution of a self-inducible cytolethal distending toxin type V-encoding bacteriophage from *Escherichia coli* O157: H7 to *Shigella sonnei*. *J. Virol.* 87 13665–13675. 10.1128/JVI.02860-13 24109226 PMC3838278

[B3] Arndt D. Marcu A. Liang Y. Wishart D. S. (2017). PHAST, PHASTER and PHASTEST: tools for finding prophage in bacterial genomes. *Brief. Bioinform.* 20 1560–1567. 10.1093/bib/bbx121 29028989 PMC6781593

[B4] Balding C. Bromley S. A. Pickup R. W. Saunders J. R. (2005). Diversity of phage integrases in *Enterobacteriaceae*: development of markers for environmental analysis of temperate phages. *Environ. Microbiol.* 7 1558–1567. 10.1111/j.1462-2920.2005.00845.x 16156729

[B5] Bonanno L. Loukiadis E. Mariani-Kurkdjian P. Oswald E. Garnier L. Michel V. (2015). Diversity of Shiga toxin-producing *Escherichia coli* (STEC) O26: H11: characterization of stx subtypes and insertion sites of Stx and EspK bacteriophages. *Appl. Environ. Microbiol.* 81 3712–3721. 10.1128/AEM.00077-15 25819955 PMC4421068

[B6] Browne A. S. Midwinter A. C. Withers H. Cookson A. L. Biggs P. J. Marshall J. C. (2018). Molecular epidemiology of Shiga toxin-producing *Escherichia coli* (STEC) on New Zealand dairy farms: application of a culture-independent assay and whole genome sequencing. *Appl. Environ. Microbiol.* 84:e481-18. 10.1128/AEM.00481-18 29752274 PMC6029106

[B7] Castillo D. Kauffman K. Hussain F. Kalatzis P. Rørbo N. Polz M. F. (2018). Widespread distribution of prophage-encoded virulence factors in marine *Vibrio* communities. *Sci. Rep.* 8:9973. 10.1038/s41598-018-28326-9 29967440 PMC6028584

[B8] CDC (2014). *E. coli (Escherichia coli).* Atlanta, GA: CDC.

[B9] CDC (2018). *Multistate Outbreak of Shiga toxin-producing Escherichia coli O157:H7 Infections Linked to Leafy Greens (Final Update).* Atlanta, GA: CDC.

[B10] CDC (2019). *Reports of Selected E. coli Outbreak Investigations.* Atlanta, GA: CDC.

[B11] Chen C. Y. Richardson J. P. (1987). Sequence elements essential for rho-dependent transcription termination at lambda tR1. *J. Biol. Chem.* 262 11292–11299. 3038914

[B12] Chen Y. Luo Y. Curry P. Timme R. Melka D. Doyle M. (2017). Assessing the genome level diversity of *Listeria monocytogenes* from contaminated ice cream and environmental samples linked to a listeriosis outbreak in the United States. *PLoS One* 12:e0171389. 10.1371/journal.pone.0171389 28166293 PMC5293252

[B13] Colello R. Cáceres M. E. Ruiz M. J. Sanz M. Etcheverría A. I. Padola N. L. (2016). From farm to table: follow-up of Shiga toxin-producing *Escherichia coli* throughout the pork production chain in Argentina. *Front. Microbiol.* 7:93. 10.3389/fmicb.2016.00093 26903972 PMC4744844

[B14] Cooper K. K. Mandrell R. E. Louie J. W. Korlach J. Clark T. A. Parker C. T. (2014). Complete genome sequences of two *Escherichia coli* O145: H28 outbreak strains of food origin. *Genome Announc.* 2:e482-14. 10.1128/genomeA.00482-14 24855308 PMC4032123

[B15] Creuzburg K. Recktenwald J. Kuhle V. Herold S. Hensel M. Schmidt H. (2005). The Shiga toxin 1-converting bacteriophage BP-4795 encodes an NleA-like type III effector protein. *J. Bacteriol.* 187 8494–8498. 10.1128/JB.187.24.8494-8498.2005 16321954 PMC1317009

[B16] Dallman T. J. Ashton P. M. Byrne L. Perry N. T. Petrovska L. Ellis R. (2015). Applying phylogenomics to understand the emergence of Shiga-toxin-producing *Escherichia coli* O157: H7 strains causing severe human disease in the UK. *Microb. Genom.* 1:e000029. 10.1099/mgen.0.000029 28348814 PMC5320567

[B17] Fang Y. Mercer R. G. McMullen L. M. Gänzle M. G. (2017). Induction of Shiga toxinencoding prophage by abiotic environmental stress in food. *Appl. Environ. Microbiol.* 83:e1378-17. 10.1128/AEM.01378-17 28778890 PMC5601330

[B18] Fortier L.-C. (2018). Bacteriophages contribute to shaping *Clostridioides (Clostridium) difficile* species. *Front. Microbiol.* 9:2033 10.3389/fmicb.2018.02033 PMC6127314 30233520

[B19] Frank C. Werber D. Cramer J. P. Askar M. Faber M. Heiden M. (2011). Epidemic profile of Shiga-toxin–producing *Escherichia coli* O104: H4 outbreak in Germany. *N. Engl. J. Med.* 365 1771–1780. 10.1056/NEJMoa1106483 21696328

[B20] Gadang V. P. Hettiarachchy N. S. Johnson M. G. Owens C. (2008). Evaluation of antibacterial activity of whey protein isolate coating incorporated with nisin, grape seed extract, malic acid, and EDTA on a turkey frankfurter system. *J. Food Sci.* 73 M389–M394. 10.1111/j.1750-3841.2008.00899.x 19019119

[B21] Grau-Leal F. Quiros P. Martinez-Castillo A. Muniesa M. (2015). Free Shiga toxin 1-encoding bacteriophages are less prevalent than Shiga toxin 2 phages in extraintestinal environments. *Environ. Microbiol.* 17 4790–4801. 10.1111/1462-2920.13053 26373580

[B22] Guy R. A. Tremblay D. Beausoleil L. Harel J. Champagne M.-J. (2014). Quantification of *E. coli* O157 and STEC in feces of farm animals using direct multiplex real time PCR (qPCR) and a modified most probable number assay comprised of immunomagnetic bead separation and qPCR detection. *J. Microbiol. Methods* 99 44–53. 10.1016/j.mimet.2014.02.002 24530484

[B23] Hammerl J. A. Göllner C. Al Dahouk S. Nöckler K. Reetz J. Hertwig S. (2016). Analysis of the first temperate broad host range brucellaphage (BiPBO1) isolated from *B. inopinata*. *Front. Microbiol.* 7:24. 10.3389/fmicb.2016.00024 26858702 PMC4729917

[B24] Imamovic L. Jofre J. Schmidt H. Serra-Moreno R. Muniesa M. (2009). Phage-mediated Shiga toxin 2 gene transfer in food and water. *Appl. Environ. Microbiol.* 75 1764–1768. 10.1128/AEM.02273-08 19168651 PMC2655461

[B25] Imamovic L. Muniesa M. (2012). Characterizing RecA-independent induction of Shiga toxin2-encoding phages by EDTA treatment. *PLoS One* 7:e32393. 10.1371/journal.pone.0032393 22393404 PMC3290563

[B26] Jäckel C. Hertwig S. Scholz H. C. Nockler K. Reetz J. Hammerl J. A. (2017). Prevalence, host range, and comparative genomic analysis of temperate *Ochrobactrum* phages. *Front. Microbiol.* 8:1207. 10.3389/fmicb.2017.01207 28713341 PMC5492332

[B27] Juska A. Gouveia L. Gabriel J. Koneck S. (2000). Negotiating bacteriological meat contamination standards in the US: the case of *E. coli* O157:H7. *Sociol. Ruralis* 40 249–271. 10.1111/1467-9523.00146

[B28] Karaman M. W. (2003). Genomes, 2nd edition. *J. Hered.* 94 432–433. 10.1093/jhered/esg082

[B29] Karlsson J. L. Cardoso-Palacios C. Nilsson A. S. Haggård-Ljungquist E. (2006). Evolution of immunity and host chromosome integration site of P2-like coliphages. *J. Bacteriol.* 188 3923–3935. 10.1128/JB.01953-05 16707684 PMC1482927

[B30] Karmali M. A. (2017). Emerging public health challenges of Shiga toxin-producing *Escherichia coli* related to changes in the pathogen, the population, and the environment. *Clin. Infect. Dis.* 64 371–376. 10.1093/cid/ciw708 27986670

[B31] Katoh K. Standley D. M. (2013). MAFFT multiple sequence alignment software version 7: improvements in performance and usability. *Mol. Biol. Evol.* 30 772–780. 10.1093/molbev/mst010 23329690 PMC3603318

[B32] Kennedy C.-A. Fanning S. Karczmarczyk M. Byrne B. Monaghan A. Bolton D. (2017). Characterizing the multidrug resistance of non-O157 Shiga Toxin-Producing *Escherichia coli* isolates from cattle farms and abattoirs. *Microb. Drug Resist.* 23 781–790. 10.1089/mdr.2016.0082 28304216

[B33] Krüger A. Burgán J. Friedrich A. W. Rossen J. W. A. Lucchesi P. M. A. (2018). ArgO145, a Stx2a prophage of a bovine O145: H-STEC strain, is closely related to phages of virulent human strains. *Infect. Genet. Evol.* 60 126–132. 10.1016/j.meegid.2018.02.024 29476813

[B34] Kumar S. Stecher G. Tamura K. (2016). MEGA7: molecular evolutionary genetics analysis version 7.0 for bigger datasets. *Mol. Biol. Evol.* 33 1870–1874. 10.1093/molbev/msw054 27004904 PMC8210823

[B35] Larrañaga O. Brown-Jaque M. Quirós P. Gómez-Gómez C. Blanch A. R. Rodríguez-Rubio L. (2018). Phage particles harboring antibiotic resistance genes in fresh-cut vegetables and agricultural soil. *Environ. Int.* 115 133–141. 10.1016/j.envint.2018.03.019 29567433

[B36] Letunic I. Bork P. (2019). Interactive Tree Of Life (iTOL) v4: recent updates and new developments. *Nucleic Acids Res.* 47 W256–W259. 10.1093/nar/gkz239 30931475 PMC6602468

[B37] Liao Y. T. Sun X. Quintela I. A. Bridges D. F. Liu F. Zhang Y. (2019). Discovery of Shiga toxin-producing *Escherichia coli* (STEC)-specific bacteriophages from non-fecal composts using genomic characterization. *Front. Microbiol.* 10:627. 10.3389/fmicb.2019.00627 31001216 PMC6454146

[B38] Łoś J. M. Łoś M. Wegrzyn G. Wegrzyn A. (2009). Differential efficiency of induction of various lambdoid prophages responsible for production of Shiga toxins in response to different induction agents. *Microb. Pathog.* 47 289–298. 10.1016/j.micpath.2009.09.006 19761828

[B39] Łoś J. M. Łoś M. Wȩgrzyn A. Wȩgrzyn G. (2010). Hydrogen peroxide-mediated induction of the Shiga toxin-converting lambdoid prophage ST2-8624 in *Escherichia coli* O157:H7. *FEMS Immunol. Med. Microbiol.* 58 322–329. 10.1111/j.1574-695X.2009.00644.x 20070366

[B40] Marder E. P. Griffin P. M. Cieslak P. R. Dunn J. Hurd S. Jervis R. (2018). Preliminary incidence and trends of infections with pathogens transmitted commonly through food—foodborne diseases active surveillance network, 10 US Sites, 2006–2017. *Morb. Mortal. Wkly. Rep.* 67 324–328. 10.15585/mmwr.mm6711a3 29565841 PMC5868202

[B41] Menouni R. Hutinet G. Petit M. A. Ansaldi M. (2015). Bacterial genome remodeling through bacteriophage recombination. *FEMS Microbiol. Lett.* 362 1–10. 10.1093/femsle/fnu022 25790500

[B42] Metzker M. L. (2010). Sequencing technologies the next generation. *Nat. Rev. Genet.* 11 31–46. 10.1038/nrg2626 19997069

[B43] Muniesa M. Blanco J. E. de Simón M. Serra-Moreno R. Blanch A. R. Jofre J. (2004). Diversity of stx2 converting bacteriophages induced from Shiga-toxin-producing *Escherichia coli* strains isolated from cattle. *Microbiology* 150 2959–2971. 10.1099/mic.0.27188-0 15347754

[B44] Neely M. N. Friedman D. I. (1998). Arrangement and functional identification of genes in the regulatory region of lambdoid phage H-19B, a carrier of a Shiga-like toxin. *Gene* 223 105–113. 10.1016/s0378-1119(98)00236-4 9858702

[B45] Nüesch-Inderbinen M. Morach M. Cernela N. Althaus D. Jost M. Mäusezahl M. (2018). Serotypes and virulence profiles of Shiga toxin-producing *Escherichia coli* strains isolated during 2017 from human infections in Switzerland. *Int. J. Med. Microbiol.* 308 933–939. 10.1016/j.ijmm.2018.06.011 30042042

[B46] Osińska A. Korzeniewska E. Harnisz M. Niestȩpski S. (2018). “The prevalence of virulence genes specific for *Escherichia coli* in wastewater samples from wastewater treatment plants with the activated sludge process,” in *E3S Web of Conferences*, Vol. 44, *EDP Sciences*, 00133 10.1051/e3sconf/20184400133

[B47] Plunkett G. Rose D. J. Durfee T. J. Blattner F. R. (1999). Sequence of Shiga toxin 2 phage 933W from *Escherichia coli* O157:H7: Shiga toxin as a phage late-gene product. *J. Bacteriol.* 181 1767–1778. 10074068 10.1128/jb.181.6.1767-1778.1999 PMC93574

[B48] Probert W. S. Miller G. M. Ledin K. E. (2017). Contaminated stream water as source for *Escherichia coli* O157 illness in children. *Emerg. Infect. Dis.* 23 1216–1218. 10.3201/eid2307.170226 28628436 PMC5512484

[B49] Quirós P. Muniesa M. (2017). Contribution of cropland to the spread of Shiga toxin phages and the emergence of new Shiga toxin-producing strains. *Sci. Rep.* 7:7796. 10.1038/s41598-017-08169-6 28798380 PMC5552810

[B50] Schmidt H. (2001). Shiga-toxin-converting bacteriophages. *Res. Microbiol.* 152 687–695. 10.1016/s0923-2508(01)01249-9 11686382

[B51] Serra-Moreno R. Jofre J. Muniesa M. (2007). Insertion site occupancy by stx2 bacteriophages depends on the locus availability of the host strain chromosome. *J. Bacteriol.* 189 6645–6654. 10.1128/JB.00466-07 17644594 PMC2045183

[B52] Sivarooban T. Hettiarachchy N. S. Johnson M. G. (2008). Physical and antimicrobial properties of grape seed extract, nisin, and EDTA incorporated soy protein edible films. *Food Res. Int.* 41 781–785. 10.1016/j.foodres.2008.04.007

[B53] Thomas R. R. Brooks H. J. L. O’brien R. (2017). Prevalence of Shiga toxin-producing and enteropathogenic *Escherichia coli* marker genes in diarrhoeic stools in a New Zealand catchment area. *J. Clin. Pathol.* 70 81–84. 10.1136/jclinpath-2016-203882 27698249

[B54] Yan Y. Shi Y. Cao D. Meng X. Xia L. Sun J. (2011). Prevalence of Stx phages in environments of a pig farm and lysogenic infection of the field *E. coli* O157 isolates with a recombinant converting phage. *Curr. Microbiol.* 62 458–464. 10.1007/s00284-010-9729-8 20697714

[B55] Yin S. Rusconi B. Sanjar F. Goswami K. Xiaoli L. Eppinger M. (2015). *Escherichia coli* O157: H7 strains harbor at least three distinct sequence types of Shiga toxin 2a-converting phages. *BMC Genomics* 16:733. 10.1186/s12864-015-1934-1 26416807 PMC4587872

[B56] Zeng H. Zhang J. Li C. Xie T. Ling N. Wu Q. (2017). The driving force of prophages and CRISPR-Cas system in the evolution of *Cronobacter sakazakii*. *Sci. Rep.* 7:40206. 10.1038/srep40206 28057934 PMC5216340

