# Supplementary figures and images for: Prediction, Diversity, and Genomic Analysis of Temperate Phages Induced From Shiga Toxin-Producing Escherichia coli Strains
Source: Front Microbiol. 2020 Jan 21;10:3093. doi: 10.3389/fmicb.2019.03093 (PMC6986202; doi:10.3389/fmicb.2019.03093)

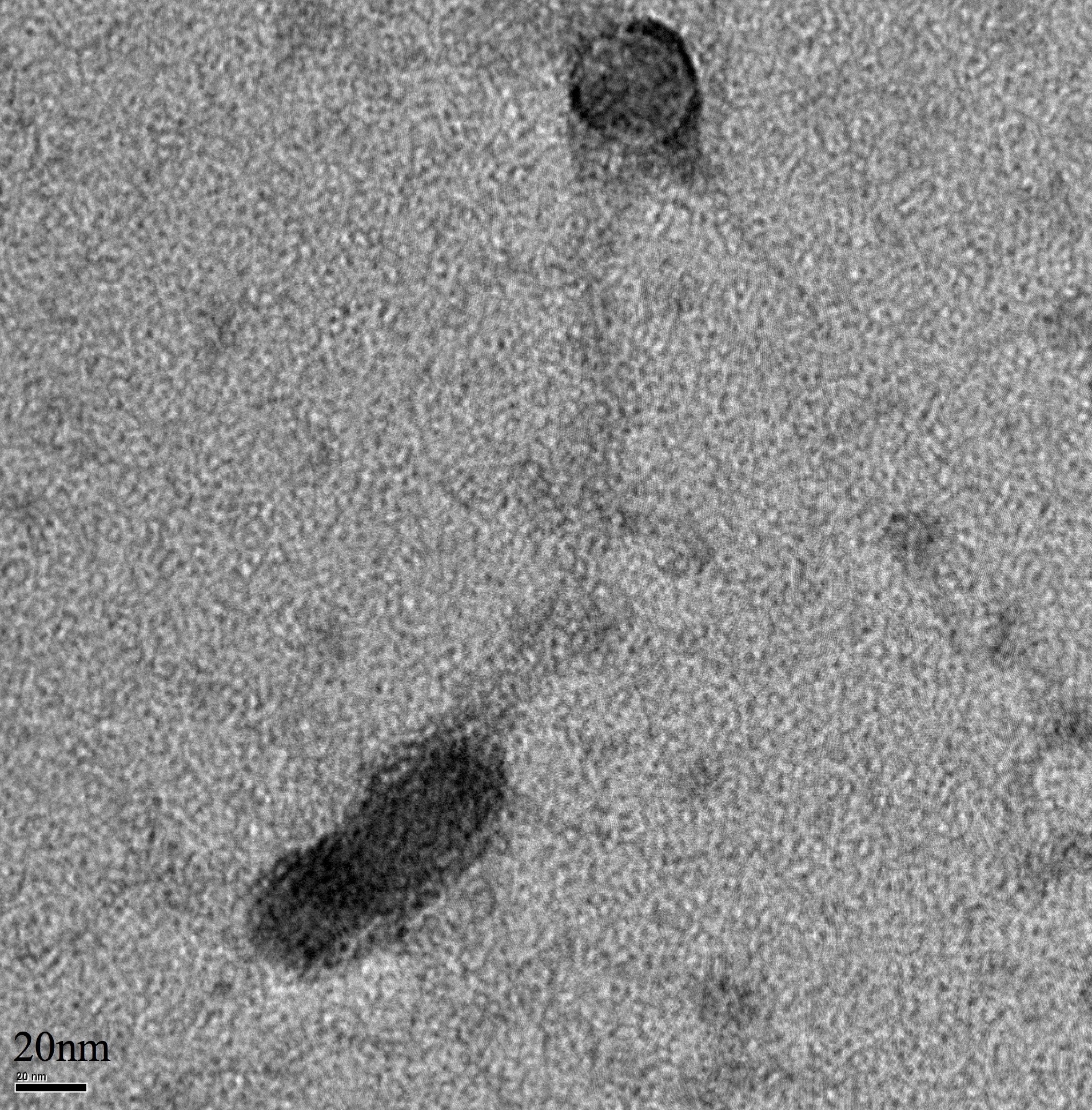

Supplement: Supplementary file 1 [file Image_1.JPEG]
