# Supplementary material for: Prediction, Diversity, and Genomic Analysis of Temperate Phages Induced From Shiga Toxin-Producing Escherichia coli Strains
Source: Front Microbiol. 2020 Jan 21;10:3093. doi: 10.3389/fmicb.2019.03093 (PMC6986202; doi:10.3389/fmicb.2019.03093)
Supplement: Supplementary file 2 [file Data_Sheet_1.docx]

Table S1. Information on different Shiga toxin-producing *E. coli* strains used for prophage prediction in this study.

| **Serotypes** | **Strain ID** | **Isolation Source** | ***Stx1*** | ***Stx2*** | **Accession Number** | **Sequencing Technology** |
| --- | --- | --- | --- | --- | --- | --- |
| O26 | RM10386 | feces | +* | + | CP028126 | PacBio-Illumina hybrid |
|  | RM8426 | creek | + | - | CP028116 | PacBio-Illumina hybrid |
|  | 2013C-3277 | stool | - | + | CP027331 | PacBio |
|  | S17-13 | fecal | - | + | CP024997 | PacBio |
|  | 11368 | /**^~^** | + | - | AP010953 | \^ |
| O45 | RM11911 | Water | + | - | CP044313 | PacBio |
|  | 2011C-4251 | / | + | + | CP027388 | PacBio |
|  | RM13745 | / | + | - | CP044312 | PacBio |
|  | RM13752 | / | + | - | CP044311 | PacBio |
|  | SJ7 | feces | + | - | CP044315 | PacBio |
| O103 | RM8385 | feces | + | - | CP028112 | PacBio-Illumina hybrid |
|  | 12009 | / | + | + | AP010958 | \ |
|  | 2015C-3163 | stool | + | - | CP027219 | PacBio |
|  | 2013C-3264 | stool | + | - | CP027544 | PacBio |
|  | 2013C-4225 | stool | + | - | CP027577 | PacBio |
| O111 | RM9975 | feces | + | - | CP028432 | PacBio-Illumina hybrid |
|  | 11128 | / | + | + | AP010960 | \ |
|  | 95JB1 | fecal | + | + | CP021335 | PacBio |
|  | 95NR1 | fecal | + | + | CP021339 | PacBio |
|  | 2015C-3101 | stool | + | + | CP027221 | PacBio |
| O121 | RM8352 | creek sediment | - | + | CP028110 | PacBio-Illumina hybrid |
|  | 16-9255 | / | - | + | CP022407 | Illumina- Nanopore hybrid |
|  | 2015C-3107 | stool | - | + | CP027317 | PacBio |
|  | 2014C-3599 | / | - | + | CP027435 | PacBio |
|  | 2014C-4423 | stool | - | + | CP027454 | PacBio |
| O145 | RM12581 | lettuce | - | + | CP007136 | PacBio-Illumina-Sanger hybrid |
|  | 95-3192 | / | - | + | CP027362 | PacBio |
|  | 2014C-3084 | stool | + | + | CP027319 | PacBio |
|  | 2015C-3125 | stool | + | + | CP027763 | PacBio |
|  | RM9872 | feces | - | + | CP028379 | PacBio-Illumina hybrid |
| O157 | EDL933 | / | + | + | CP008957 | PacBio-Illumina hybrid |
|  | pv15-279 | / | + | + | AP018488 | PacBio-Illumina hybrid |
|  | EC4115 | / | - | + | CP001164 | / |
|  | TW14359 | / | - | + | CP001368 | / |
|  | SS17 | / | - | + | CP008805 | Ion Torrent |
| Others | RM9245 | feces | - | + | CP044314 | PacBio |
|  | 2011C-3493 | / | - | + | CP003289 | \ |
|  | FDAARGOS_403 | / | - | + | NZ_CP023535 | PacBio-Illumina hybrid |
|  | SMN197SH3 | cattle | - | + | NZ_CP024056 | PacBio-Illumina hybrid |
|  | HUSEC2011 | / | - | + | NZ_HF572917 | \ |

* The presence of the *stx1* gene and the *stx2* gene (+ or -) were provided by the annotation of whole-genome sequences.

**^~^**specific sources not available.

^ The sequencing technology not available. The sequencing technology means the genome assemblies were obtained through certain sequencing platform indicated on NCBI database.

Table S2. Oligonucleotides for screening and quantifying the *stx* genes used in this study. PCR: conventional PCR; qPCR: quantitative real-time PCR; up: upper primer; lp: lower primer; FAM: 6-carboxyfluorescein reporter; 3IABKFQ: dark quencher.

| **Target gene** | **Reaction** | **Oligonucleotide** | **Sequence** | **Reference** |
| --- | --- | --- | --- | --- |
| *Stx1* | PCR | up | CATCGCGAGTTGCCAGAATG | (Liao *et al.*, 2018) |
|  |  | lp | AATTGCCCCCAGAGTGGATG |  |
|  | qPCR | up | GCGGTTACATTGTCTGGTGACA | (Grau-Leal *et al.*, 2015) |
|  |  | lp | GCATCCCCGTACGACTGATC |  |
|  |  | probe | /56FAM/TAGCTATAC/ZEN/CACGTTACAGCG/3IABKFQ/ |  |
| *Stx2* | PCR | up | GTATACGATGACGCCGGGAG | (Liao *et al.*, 2018) |
|  |  | lp | TTCTCCCCACTCTGACACCA |  |
|  | qPCR | up | ACGGACAGCAGTTATACCACTCT | (Imamovic *et al.*, 2010) |
|  |  | lp | CTGATTTGCATTCCGGAACGT |  |
|  |  | probe | /56-FAM/CCAGCGCTG/ZEN/CGACACG/3IABKFQ/ |  |

Grau-Leal, F., Quiros, P., Martinez-Castillo, A., and Muniesa, M. (2015) Free Shiga toxin 1-encoding bacteriophages are less prevalent than Shiga toxin 2 phages in extraintestinal environments. Environ Microbiol 17: 4790–4801.

Imamovic, L., Serra-Moreno, R., Jofre, J., and Muniesa, M. (2010) Quantification of Shiga toxin 2-encoding bacteriophages, by real-time PCR and correlation with phage infectivity. J Appl Microbiol.

Liao, Y.-T., Quintela, I.A., Nguyen, K., Salvador, A., Cooley, M.B., and Wu, V.C.H. (2018) Investigation of prevalence of free Shiga toxin-producing Escherichia coli (STEC)-specific bacteriophages and its correlation with STEC bacterial hosts in a produce-growing area in Salinas, California. PLoS One 13: e0190534.

Figure S1. Morphology of Stx1-converting phages Lys8385Vzw with two capsids and a long tail observed by transmission electron microscopy.
